# Evaluation of suitable reference genes for qRT-PCR normalization in strawberry (*Fragaria *×* ananassa*) under different experimental conditions

**DOI:** 10.1186/s12867-018-0109-4

**Published:** 2018-06-22

**Authors:** Yunting Zhang, Xiaorui Peng, Yi Liu, Yali Li, Ya Luo, Xiaorong Wang, Haoru Tang

**Affiliations:** 1 College of Horticulture, Sichuan Agricultural University, Chengdu, 611130 China; 2 Institute of Pomology and Olericulture, Sichuan Agricultural University, Chengdu, 611130 China

**Keywords:** Reference gene, Normalization, qRT-PCR, Gene expression, Strawberry

## Abstract

**Background:**

Strawberry has received much attention due to its nutritional value, unique flavor, and attractive appearance. The availability of the whole genome sequence and multiple transcriptome databases allows the great possibility to explore gene functions, comprehensively. Gene expression profiles of a target gene can provide clues towards the understanding of its biological function. Quantitative real-time PCR (qRT-PCR) is a preferred method for rapid quantification of gene expression. The accuracy of the results obtained by this method requires the reference genes with consistently stable expression to normalize its data.

**Results:**

In present study, the expression stability of seven candidate reference genes in diverse sample subsets of different tissues and fruit developmental stages, and plant subjected to light quality and low temperature treatments was evaluated using three statistical algorithms, geNorm, NormFinder, and BestKeeper. Our data indicated that the expression stability of reference genes varied under different experimental conditions. Overall, *DBP*, *HISTH4*, *ACTIN1* and *GAPDH* expressed much more stably. *PIRUV*, *ACTIN2* and *18S* were not recommended for normalization in given experimental conditions due to low stability. In addition, the relative expression pattern of *HY5* (ELONGATED HYPOCOTYL5) was conducted to further confirm the reliability of the reference genes, which demonstrated the correct adoption of reference genes was of great importance in qRT-PCR analysis.

**Conclusions:**

Expression stability of reference genes from strawberry varied across selected experimental conditions. Systematic validation of reference genes prior to calculation of target gene expression level should be done to improve the accuracy and consistency of qRT-PCR analysis.

**Electronic supplementary material:**

The online version of this article (10.1186/s12867-018-0109-4) contains supplementary material, which is available to authorized users.

## Background

Gene expression analysis reflects the quantification of mRNA transcription levels of selected genes and provides a novel insight into the genes function in signaling transduction, metabolic mechanism and regulatory network. Quantitative real-time PCR (qRT-PCR) has become a very prevalent technique to determine the level of gene expression and validate the results of high-throughput array experiments and transcriptomes, due to its rapidity, specificity, sensitivity and reproducibility [1]. However, the accuracy of qRT-PCR is severely affected by many variations including the quality and quantity of mRNA templates, reverse transcription of mRNA, amplification efficiency of primers, genotype of the samples, different tissues and developmental stages, abiotic and biotic stress, etc. [2–4]. Thus, a normalization step is an essential prerequisite to minimize the effect of these variations. Currently, the most general approach for qRT-PCR normalization is the application of reference genes, but the use of inappropriate reference genes can cause significant deviation and misinterpretations of data, finally leading to imprecise, even erroneous results [5, 6]. An ideal reference gene should keep the expression at a constant level under various conditions and cannot be influenced by experimental parameters. Unfortunately, no absolute universal reference gene has been identified in plants or animals. Many previous studies directly selected the traditional reference genes involved in basic cellular process, primary metabolism and cell structure maintenance such as 18S ribosomal RNA (18S rRNA), tubulin (TUB), ubiquitin (UBQ), actin (ACT) and glyceraldehyde-3-phosphate dehydrogenase (GAPDH) to standardize the target genes for qPCR gene expression without evaluating the expression stability. Yet, facts have proved that these reference genes do not always show perfectly stable expression in response to a variety of conditions [7–9]. Besides, some researches pointed out the use of two or more should be demanded when a single reference gene cannot meet the experimental requirements [10, 11]. Hence, selection and validation of reference genes must be conducted in each experimental background prior to their use in qRT-PCR normalization analysis.

Strawberry is one of the favorite fruit crops and widely consumed throughout the world with its pleasant aroma, flavor, taste and texture. Especially, it is rich in human health-promoting nutrients such as vitamins, minerals, antioxidant compounds and thus considered as the functional food, which receives more and more attention from plant breeder, food industry and consumer [12–14]. These characteristics of strawberry involve in complex metabolism processes regulated by both internal and external factors that can affect the related gene expression. Therefore, studies of expression patterns may supply helpful information to reveal the molecular mechanisms regarding genetic background, biotic and abiotic stress, contributing to development of genetic engineering strategies. Accordingly, stable reference genes are urgently needed to identify for obtaining accurate, reliable and high precision gene expression analysis data. Recently, validation of reference genes in strawberry has been only reported in limited cultivars and experimental conditions [15–17].

In this study, seven candidate reference genes were selected and assessed by qRT-PCR in different tissues, fruit at seven developmental stages, fruit treated with light quality and low temperature. Furthermore, three different statistical algorithms including geNorm [18], NormFinder [19] and Best-Keeper [20] were used to calculate the variability of the expression of the candidate genes and obtained the appropriate reference genes for normalization of gene expression under given conditions in strawberry.

## Results

### Amplification specificity and efficiency of candidate reference genes

**Fig. 1:**
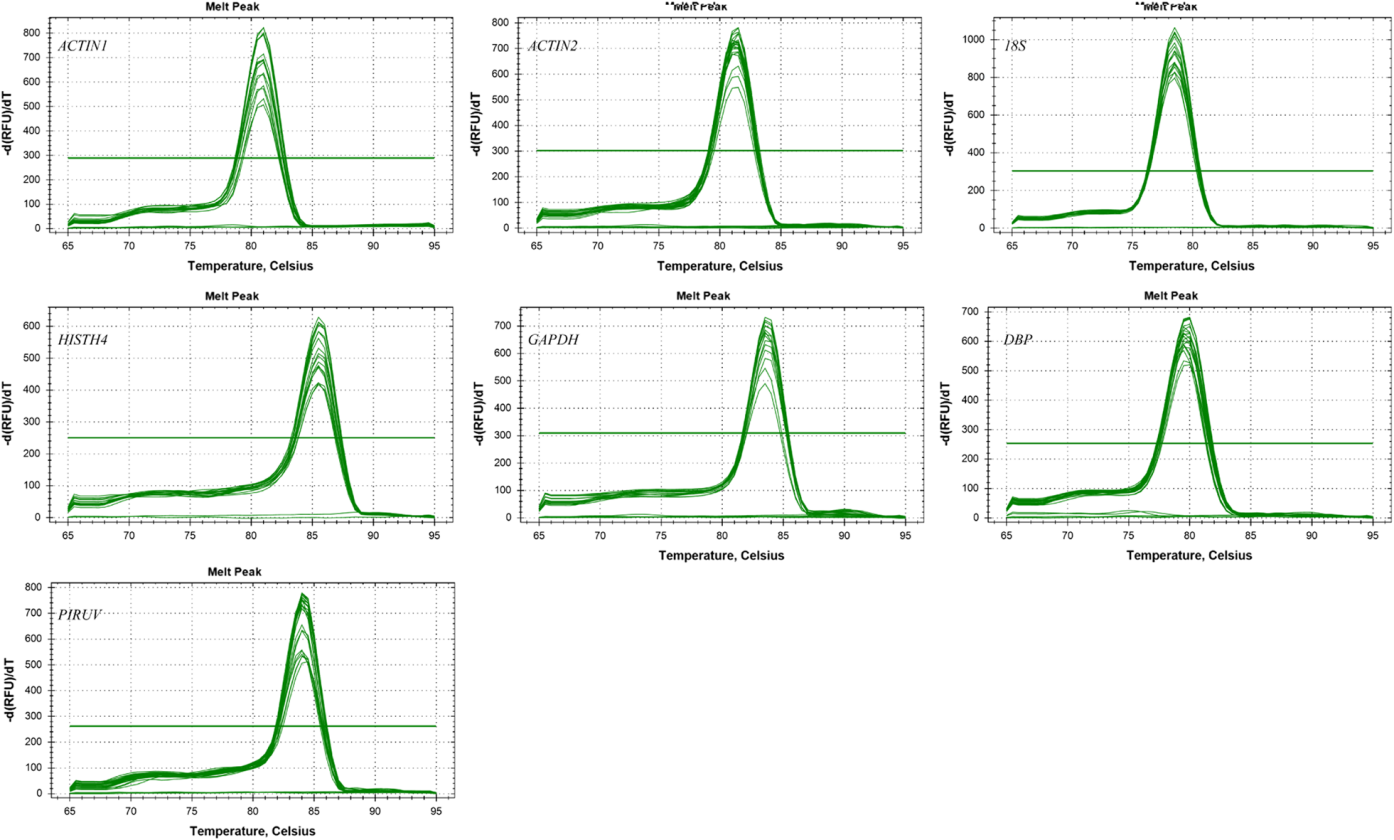
Melting curve analysis of seven candidate reference genes by quantitative real-time RT-PCR. A single peak indicated the specificity of primers

The specific primers of seven candidate reference genes were chosen for qRT-PCR, with the amplicon length ranging from 72 to 219 bp. Six primer pairs previously used as the control genes in strawberry were selected; The *ACTIN1* primers were designed using Primer Premier 5.0 software and the specificity of the amplicon was confirmed by a single PCR product presence of the expected size on 2.0% agarose gel following electrophoresis (Additional file 1: Figure S1). In addition, the presence of a single peak in the melting curve analysis showed the amplification specificity of all primers (Fig. 1). Meanwhile, no signals were detected in the no-template controls. qRT-PCR amplification efficiencies varied from 86% (*18S*) to 108.6% (*DBP*), and linear correlation coefficients (R^2^) of the standard curve ranged from 0.990 for *PIRUV* to 0.999 for *HISTH4* (Table 1).

**Table 1:** Description of seven candidate reference genes, primer sequences, and amplicon characteristics

Gene name	Accession number	Primer sequence (5′-3′) forward/reverse	Amplicon size (bp)	Annealing Tm (°C)	Melting Tm (°C)	E (%)	R^2^	References
*ACTIN1*	LC017712.1	TTCACGAGACCACCTATAACTC GCTCATCCTATCAGCGATT	122	55	81	100.3	0.994	–
*ACTIN2*	AB116565.1	GCTAATCGTGAGAAGATGAC AGCACAATACCAGTAGTACG	119	55	81.5	97.8	0.998	[21]
*18S*	X15590.1	TGTGAAACTGCGAATGGCTCATTAA GAAGTCGGGATTTGTTGCACGTATT	110	60	78.5	86	0.998	[15]
*HISTH4*	AB197150.1	TCAAGCGTATCTCCGGTCTC AGTGTCCTTCCCTGCCTCTT	163	60	85.5	92.9	0.999	[22]
*GAPDH*	AB363963.1	GAGTCTACTGGAGTGTTCA CTTGTATTCGTGCTCATTCA	135	55	83.5	104.8	0.998	[23]
*DBP*	XM_004291635.2	TTGGCAGCGGGACTTTACC CGGTTGTGTGACGCTGTCAT	72	60	80	108.6	0.995	[24]
*PIRUV*	AF141016.2	AGGTGCGTTGCGAAGAGGA CTAAATCTGTGAATGCGAATGAGG	219	60	84.5	103.5	0.990	[15]

### Expression profiles of candidate reference genes

**Fig. 2:**
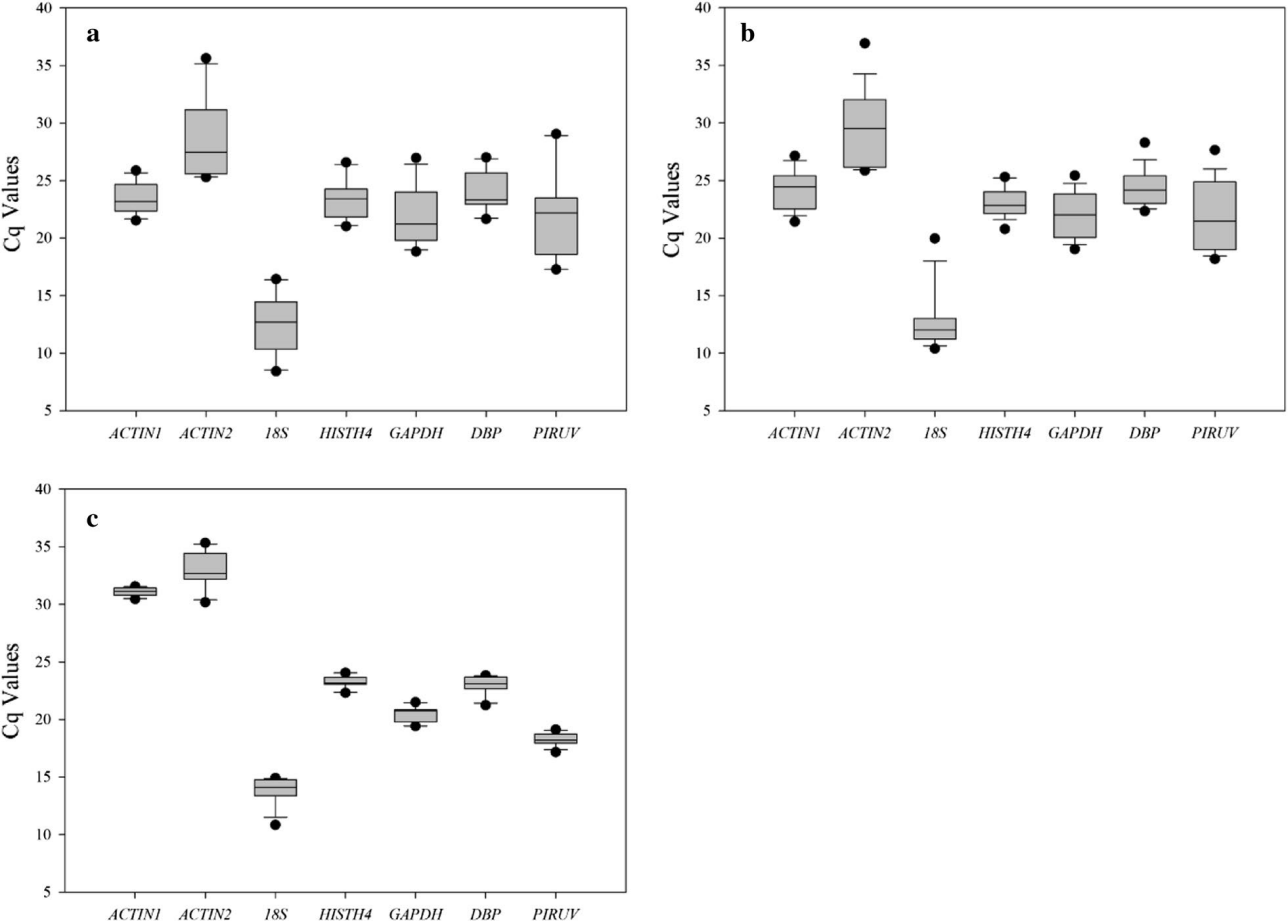
Expression profiles of seven candidate reference genes in strawberry samples. Expression data for different tissues and fruit developmental stages (**a**), fruit treated with light quality (**b**) and fruit treated with low temperature (**c**) are displayed as Cq values for each reference gene. The box indicates the 25th and 75th percentiles. A line across the box represents the median. Whiskers are depicted as the maximum and minimum values and the black circles represent outliers. The higher boxes and whiskers mean the greater variations

The quantification cycle (Cq) values in the qRT-PCR reactions are defined as the amplification cycle numbers at which the fluorescent signal reaches above the baseline threshold and are used to identify the differences in transcript expression levels. In this study, Cq-values provided an overview of the gene expression levels of seven candidate reference genes in the three subset samples from different tissues and fruit developmental stages, light quality treatment, and low temperature treatment, respectively (Fig. 2). The results showed the *18S* was the most expressed gene in three subsets, with the lowest mean Cq value (12.37, 12.81 and 13.85, respectively). By contrast, *ACTIN2* was the least expressed gene with the highest mean Cq value in three subgroups (28.65, 29.47 and 32.88, respectively). Additionally, *ACTIN2*, *PIRUV* and *18S* displayed a larger expression variation among the evaluated genes in all subsets according to the size of the boxes and whisker tapes, while *HISTH4*, *DBP* and *GAPDH* had a smaller variation and expressed moderately. Interestingly, Cq values of *ACTIN1* were intensive within a group, whereas the data (mean Cq = 31.09) in “low temperature” subset was much higher than other two subsets. The wide expression ranges of the seven tested reference genes demonstrated that no single reference gene had a constant expression in different samples. Therefore, it is of utmost importance to select a reliable reference gene to normalize gene expression under a certain condition.

### Expression stability analysis of candidate reference genes

The expression stability of seven candidate reference genes across different experimental sets of samples was determined and ranked using three different computational algorithms including NormFinder, geNorm and BestKeeper, as shown in Table 2.

**Table 2:** Stability ranking of seven candidate reference genes by geNorm, NormFinder, and BestKeeper

Group	Rank	geNorm		NormFinder		BestKeeper	
		Gene	Stability	Gene	Stability	Gene	SD dev [± CP]
Different tissues and fruit developmental stages	1	*DBP*	0.96	*GAPDH*	0.291	*ACTIN1*	0.98
	2	*HISTH4*	0.96	*HISTH4*	0.352	*HISTH4*	1.41
	3	*GAPDH*	1.07	*DBP*	0.443	*DBP*	1.57
	4	*18S*	1.24	*18S*	0.699	*GAPDH*	1.94
	5	*ACTIN1*	1.54	*ACTIN1*	0.781	*18S*	2.22
	6	*ACTIN2*	1.87	*ACTIN2*	1.036	*ACTIN2*	2.62
	7	*PIRUV*	2.20	*PIRUV*	1.211	*PIRUV*	2.68
Light quality	1	*GAPDH*	0.72	*GAPDH*	0.251	*HISTH4*	1.05
	2	*DBP*	0.72	*DBP*	0.335	*DBP*	1.30
	3	*ACTIN1*	0.85	*ACTIN1*	0.347	*ACTIN1*	1.45
	4	*HISTH4*	1.00	*HISTH4*	0.810	*GAPDH*	1.67
	5	*18S*	1.24	*18S*	1.034	*18S*	1.81
	6	*ACTIN2*	1.51	*ACTIN2*	1.286	*PIRUV*	2.60
	7	*PIRUV*	1.75	*PIRUV*	1.501	*ACTIN2*	2.84
Low temperature	1	*ACTIN1*	0.56	*HISTH4*	0.190	*ACTIN1*	0.29
	2	*HISTH4*	0.56	*ACTIN1*	0.211	*HISTH4*	0.38
	3	*PIRUV*	0.67	*PIRUV*	0.438	*PIRUV*	0.38
	4	*GAPDH*	0.74	*DBP*	0.501	*DBP*	0.58
	5	*DBP*	0.78	*GAPDH*	0.544	*GAPDH*	0.58
	6	*18S*	0.99	*18S*	0.864	*18S*	0.82
	7	*ACTIN2*	1.18	*ACTIN2*	1.041	*ACTIN2*	1.17
Total	1	*DBP*	0.98	*HISTH4*	0.243	*HISTH4*	0.95
	2	*HISTH4*	0.98	*GAPDH*	0.307	*DBP*	1.25
	3	*GAPDH*	1.08	*DBP*	0.406	*GAPDH*	1.54
	4	*ACTIN1*	1.20	*ACTIN1*	0.472	*18S*	1.78
	5	*18S*	1.44	*ACTIN2*	0.592	*PIRUV*	2.74
	6	*ACTIN2*	1.67	*18S*	0.672	*ACTIN2*	2.91
	7	*PIRUV*	1.91	*PIRUV*	0.853	*ACTIN1*	3.18

In geNorm analysis, the genes were ranked according to the average expression stability (M) values. The M-value is based on the average pair-wise variation of a certain gene with all other tested reference genes through serially exclusion of the least stably expressed genes. The cut-off range of stability value (M) is < 1.5, so the reference gene with the lowest M value was considered as the most stable expression and vice versa. When all samples from different tissues and fruit developmental stages were taken together, *DBP* and *HISTH4* had the lowest average expression stability value (M = 0.96), whereas *PIRUV* had the highest M value, indicating *DBP* and *HISTH4* were the most stably expressed, and *PIRUV* was the most variably expressed. *GAPDH* and *DBP* in samples collected from different light quality treatment showed the most stable expression and once again, *PIRUV* was the least stable gene. In the subset of samples under low temperature stress, all candidates with M value < 1.5 could be chosen as reference genes. Additionally, *ACTIN1* and *HISTH4* showed the most stable expression with the lowest M value. A combination of all samples for geNorm analysis showed that *DBP* and *HISTH4* (M = 0.98) featured the most stable expression whereas the most variable genes included *PIRUV* (M = 1.91) and *ACTIN2* (M = 1.67).

**Fig. 3:**
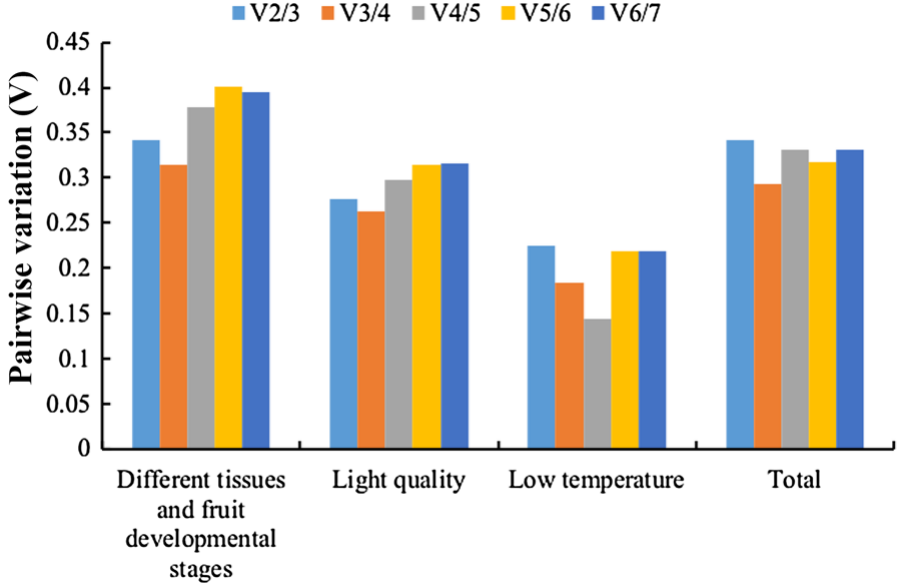
Pairwise variation (V) for determination of optimal number of reference genes for qRT-PCR normalization. Pairwise variation (V_n_/V_n+1_) was analyzed between the normalization factors NF_n_ and NF_n+1_ by the geNorm software. The optimal number of genes for normalization was determined based on V less than 0.15

The geNorm programme was also used to determine the optimal number of reference genes required for accurate normalization across the experimental conditions by calculating the pairwise variation (V_n_/V_n+1_) between two sequential normalization factors. A large pairwise variation (the recommended cut-off value ≥ 0.15) means that the added gene had a significant effect on the normalization and should preferably be included in calculation of a reliable normalization factor, whereas the V_n_/V_n+1_ value below 0.15 suggested that an extra reference gene is not required for normalization. In our study, the pairwise variation of V4/5 in low temperature treatment subset was lower than 0.15, which suggested that four genes were necessary for more reliable normalization of target genes. However, all the V-values in other three subsets were higher than 0.15 (Fig. 3). It was reported that application of multiple reference genes will probably increase the experimental instability and complexity [25]. Hence, one reference gene can also be used to perform an accurate normalization.

The stability value of the reference gene was further analyzed by NormFinder algorithm that ranks the candidates based on the evaluation of both intra- and inter-group variation and a separate analysis of the sample subgroups in expression levels. The outputs of NormFinder analysis in our study showed that the stability ranking of the seven candidate genes was relatively consistent with the data array of geNorm. *GAPDH* was the most stable genes with the minimum stability value of 0.291 and 0.251 in the first two subgroup samples, respectively, while *PIRUV* and *ACTIN2* were the least stable genes. *HISTH4* showed the greatest stability of expression in “low temperature” and “total” subsets. *ACTIN2* and *18S* showed the most variable expression with the highest stability value in “low temperature”, suggesting that they were least stable genes in that experimental conditions. *PIRUV* and *18S* were the worst stably expressed genes when all samples were combined to consider.

BestKeeper applet is another Excel-based tool to estimate the stability of a candidate reference gene according to the standard deviation (SD) of the Cq values. The lower SD-value means the gene is more stable. BestKeeper ranked *ACTIN1* as the most stable reference gene in “different tissues and fruit developmental stages” and “low temperature” subsets. *HISTH4* was found to perform better than other genes in “light quality” and “total” subsets. Furthermore, *18S*, *PIRUV* and *ACTIN2* identified in the subgroups always showed more unstable, which was consistent with the outcome from NormFinder and geNorm analysis.

### Validation of the selected reference genes

**Fig. 4:**
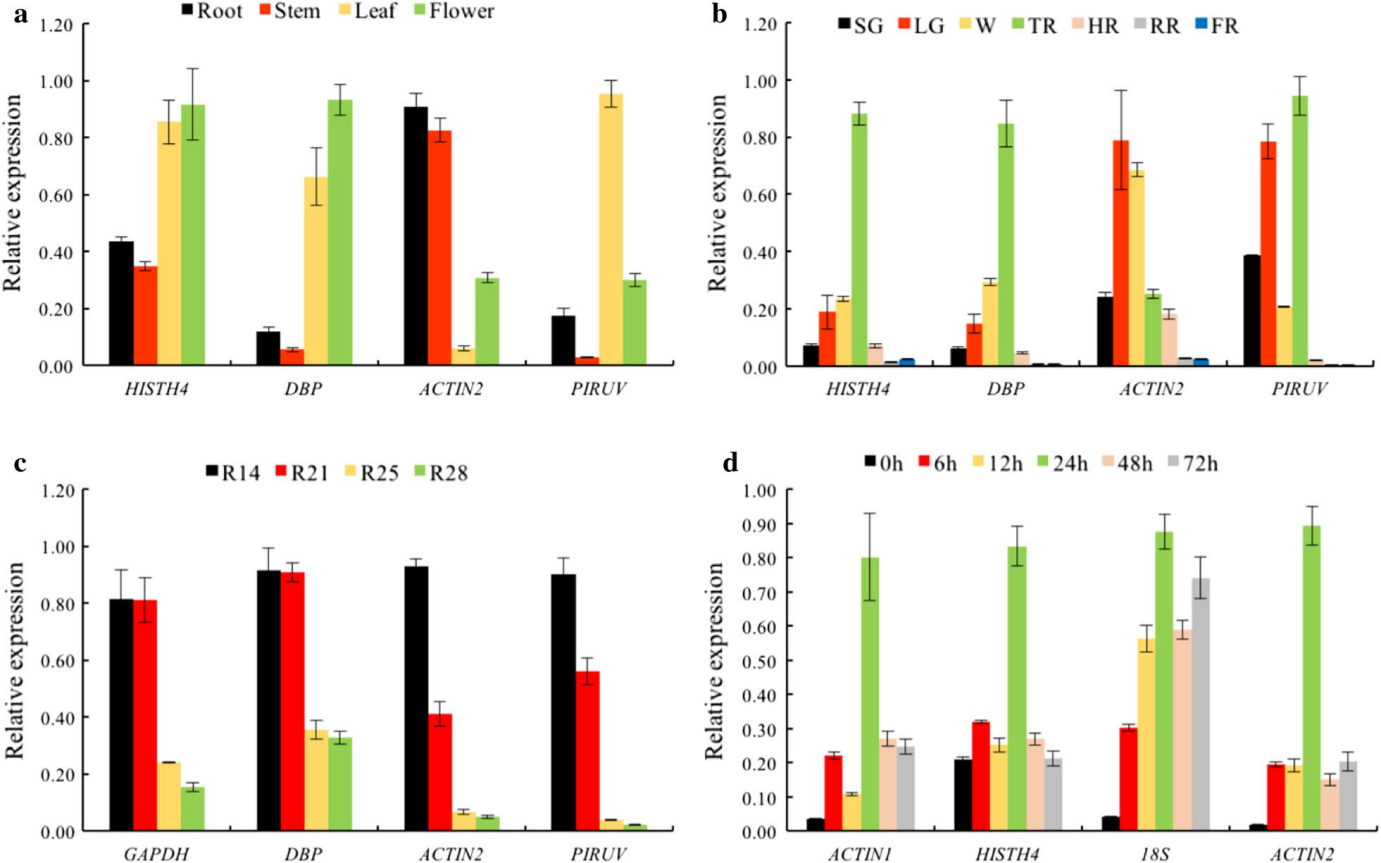
Relative quantification of *FaHY5* expression using validated reference genes for normalization in given experimental conditions. These experimental series include **a** different tissues, **b** different fruit developmental stages, **c** fruit treated with red light **d** fruit treated with low temperature. *SG* small green, *LG* large green, *W* white, *TR* turning red, *HR* half red, *RR* red ripe, *FR* full red. The relative expression levels are depicted as the mean ± standard error, which was calculated from three biological replicates

To validate the reliability of tested reference genes in some specific experiments. The relative expression levels of *FaHY5* were evaluated by selecting two most and least stable reference genes as calibrators, respectively (Fig. 4). In different tissues, the highest expression level of *FaHY5* was detected in flower, followed by leave and root, and then in stem when using the most stable reference genes (*HISTH4*, *DBP*). However, there was a distinct bias when using the most unstable reference genes (*ACTIN2*, *PIRUV*) (Fig. 4a). We observed that *FaHY5* always expressed highly before fruit coloration among all reference genes, but the change tendency normalized by the most stable references (*HISTH4*, *DBP*) and the least stable references (*HISTH4*, *DBP*) obviously differed (Fig. 4b). The *FaHY5* expression level decreased regularly along with red light treatment (R14 > R21 > R25 > R28) in fruit when *GAPDH*, *DBP*, *ACTIN2* and *PIRUV* were used as the internal control genes, if ignoring the statistical significance (Fig. 4c). The normalization outcomes of the relative expression of *FaHY5* in strawberry leaves were consistent when using the two most stable genes (*ACTIN1* and *HISTH4*) as calibrators during the low temperature treatment, while the main discrepancies were presented during normalization of the worst reference genes, *18S* and *ACTIN2* (Fig. 4d). These results demonstrated that validation of reference genes with stable expression across the experimental conditions is important for accurate normalization of target gene expression.

## Discussion

Gene expression analysis could lead to a better understanding of gene functions. Quantitative real- time PCR (qRT-PCR) is currently one of the most sensitive tool for rapid quantification of target gene expression and could ensure the data to be reliable using a valid reference gene as the internal control. The optimal reference gene used for normalization in qRT-PCR assays should be steadily transcribed at any conditions. However, such a perfect reference gene does probably not exist, because the transcript level of reference genes may vary among different species, varieties, tissues, abiotic and biotic stress, etc. For example, *18S* showed high stability in rice and virus-infected cereals [26, 27], but ranked poorly in *A. belladonna* and *Lilium* [28, 29]. *GAPDH* was found to be homogeneously expressed in grapevine and coffee [30, 31], but had a very unstable expression in peach and wheat [11, 32]. Therefore, reference genes must be validated before gene expression study to ensure accuracy and precision of results under certain experimental conditions.

In this study, seven candidate reference genes, i.e., *ACTIN1*, *ACTIN2*, *DBP*, *GAPDH*, *HISTH4* and *18S*, were assessed to improve the relative quantification by qRT-PCR for gene expression analysis in strawberry under different experimental subsets including “different tissues and fruit developmental stages”, “light quality” and “low temperature” treatments. Results suggested the primers had quite good specificity as a single peak presented in the melting curve analysis (Fig. 1). Furthermore, qRT-PCR for each tested reference gene showed high efficiency (close to 100%) except *18S* (86%) (Table 1). Observations mentioned above indicated our primers were reliable for analyses of reference gene stability.

Three algorithms, geNorm, NormFinder and BestKeeper are often used to select reference genes through evaluating their stability. Hence, divergence existed in the stability ranking most likely due to different statistical algorithms and analytical procedures for each programme. For instance, in “different tissues and fruit developmental stages” group, *HISTH4*, *DBP* and *ACTIN1* were ranked first in geNorm, Normfinder and BestKeeper, respectively. In “light quality” group, *GAPDH* was the most stable gene through geNorm, Normfinder evaluation, while it was ranked fourth by BestKeeper. In “low temperature” group, *ACTIN1*, *HISTH4* and *PIRUV* were all ranked in top three in three subsets, but *ACTIN1* and *HISTH4* exchanged their position in NormFinder analysis. *HISTH4* and *PIRUV* as the reference genes performed well when strawberry fruit responded to salt and drought stress [15], while *HISTH4* and *ACTIN* showed the best expression stability in strawberry fruits treated with benzothiadiazole resistance inducers [22]. As listed in Table 2, *ACTIN2*, *PIRUV* and *18S* always occupied the bottom position, suggesting that they were not suitable for normalization across the given experimental conditions, which can also be concluded from expression profiles (Fig. 2). During qRT-PCR analysis, suitable expression abundance and stable expression profile for selecting a valid reference gene are reliable precondition [33, 34]. However, *ACTIN2*, *PIRUV* and *18S* in our study showed a large variation on expression levels. *ACINT2* had a very low expression abundance. On the contrary, *18S* expressed quite excessively, which was not reliable for normalization of target genes with middle or low expression levels. This trait of *18S* was also presented in the study of validation of reference genes in strawberry fruits regarding different cultivars and osmotic stresses, and it was concluded as the most unstable gene in all tested conditions [15]. Previously, the *18S rRNA* gene was considered as an ideal internal control in qRT-PCR analysis [9, 35, 36], but recently it has been discarded from stable reference genes due to its extremely high expression abundance and variation in many plants [33, 37, 38]. Therefore, *18S* was not recommended as an appropriate reference gene in strawberry, although it was more stable in fruit treated with COA (a mixture of calcium and organic acids) and chitosan [22].

ELONGATED HYPOCOTYL5 (HY5), a member of the bZIP transcription factor family [39], regulates photomorphogenesis positively [40]. In addition to the role of HY5 in plant growth and development. HY5 is involved in pigment accumulation and considered as the integrator in several signaling pathway such as hormone, nutrient, abiotic and biotic stress [41]. To further validate the reliability of a reference gene, the expression level of *FaHY5* was normalized using two most and least stable reference genes in each subset (Fig. 4). the normalization results of *FaHY5* gene were more consistent when reference genes with stable expression were used as internal controls, whereas the significant differences of normalization were caused by the most variable genes, which demonstrated the correct adoption of reference genes was of great importance in qRT-PCR analysis.

## Conclusion

Failure to apply appropriate references genes for normalization in qRT-PCR always causes fallacious results. Thus, systematic validation of reference genes prior to calculation of target gene expression level should be done to improve the accuracy and consistency of qRT-PCR analysis. Our study demonstrated that the expression stability of reference genes from strawberry varied across selected experimental conditions. Apparently, the *ACTIN2*, *PIRUV* and *18S* were frequently presented as unstable genes and ranked at the bottom position by the three applications. Moreover, *18S* could be excluded from analysis without hesitation as a result of very high abundance.

## Methods

### Plant materials and treatments

Strawberry ‘Toyonoka’ was used to analyze the expression stability of candidate reference genes and grown in 15 cm × 13 cm pots filled with 2:2:1 (v/v/v) mixture of nutrient soil, garden soil and perlite, and subjected to routine management in the greenhouse of Sichuan Agricultural University. Specific tissues (roots, stems, leaves, flowers) and different developmental stages of fruits (small green, SG; large green, LG; white, W; turning red, TR; half red, HR; red ripe, RR; full red, FR) were collected for evaluation of reference genes. Simultaneously, the potted strawberries were subject to low temperature treatment at white stage of fruit. Two groups of plants were grown at 4 °C (cold) and 25 °C (control), respectively, for 0, 6, 12, 24, 48, and 72 h under a 16 h diurnal light cycle at 100 μmol m^−2^ s^−1^ with 75% relative humidity.

To select the suitable reference genes that could normalize qRT-PCR data in strawberries treated with different light quality. The potted strawberries ‘Toyonoka’ at the 7th day after flowering were divided into four groups, respectively and transferred into assigned growth chambers with controlled environmental conditions (8 h dark at 16 °C, 16 h photoperiod at 25 °C and 75% relative humidity). White (control), red (730 nm), blue (450 nm), mixed light (red: blue = 1:1) light-emitting diodes (LEDs) placed at the top of chambers were applied to irradiate strawberry seedlings with 100 μmol m^−2^ s^−1^ until fruit harvesting. Fruits were collected at the 14th, 21th, 25th, 28th day after flowering.

### Total RNA extraction and first strand cDNA synthesis

Total RNA was isolated from collected samples through the improved CTAB (hexadecyltrimethylammonium bromide) method [42]. After quality assessment and quantity determination, 1 μg of total RNA was reverse transcribed into the complementary DNA (cDNA) using PrimeScript™ RT reagent Kit with gDNA Eraser (Perfect Real Time) (Takara, Japan) according to the manufacturer’s instructions. cDNAs were diluted tenfold to be used in the quantitative real-time RT-PCR reactions (qRT-PCR).

### Selection of candidate reference genes, primer design and validation

A total of seven candidate reference genes were evaluated. These genes were chosen based on their previous use in strawberry including actin (ACT), 18S ribosomal RNA (18S rRNA), glyceralde-hyde-3-phosphate dehydrogenase (GAPDH), DNA binding protein (DBP) and histone H4 (HISTH4) and pyruvate decarboxylase (PIRUV). Primers shown in Table 1 were designed by Primer Premier 5.0 software or introduced from published papers. Before qRT-PCR, each primer pair was screened online via Primer-BLAST and then checked the hairpins, dimer formation, and amplicon specific-size.

### qRT-PCR with SYBR green

qRT-PCR was conducted on the CFX96 real-time PCR system (Bio-Rad, USA). All reactions were performed using SYBR Green Premix Ex Taq TM (Takara, Japan) in triplicate of each sample. Total 10 μl reaction volume contained 0.4 μl each primer (0.4 μM), 5 μl SYBR Premix (Takara, Japan) and 1 μl diluted cDNA template and 3.2 μl of RNase-free water. Reaction protocol was set with three-step cycling conditions: 95 °C for 3 min, followed by 40 cycles of 95 °C for 10 s, 60 °C for 30 s and 72 °C for 15 s. Melting curve was inserted, ramping from 65 °C to 95 °C (increment 0.5 °C/5 s) to verify specificity of primer amplification based on the presence of a single and sharp peak. Controls without template were included in each run to check the potential reagent contamination. Besides, for each gene, the full sample under the same experimental treatment was run on one plate to avoid any technical variation and inter-plate differences. Amplification efficiency (E) and correlation coefficient (R^2^) were tested by a standard curve based on different dilutions of the cDNA template.

### Data analysis

Expression levels of the tested seven reference genes were determined by the number of amplification cycles (Cq) at which a specific threshold level was detected. Three different Microsoft Excel-based common softwares (BestKeeper, geNorm, and NormFinder) were used to evaluate the expression stability of candidate reference genes. BestKeeper could analyze the raw Cq values directly and the candidate reference gene with the lowest standard deviation (SD) is considered as the most stable gene. geNorm and NormFinder calculations need Cq values to be converted into relative quantities according to the formula: $2^{{ - \Delta {\text{Ct}}}} \left( {\Delta {\text{ Ct}} = {\text{the}}\,{\text{corresponding}}\,{\text{Cq}}\;{\text{value}}{-}{\text{minimum}}\,{\text{Cq}}} \right).$ In geNorm, the reference gene with the lowest pairwise variation is the most stable, while in NormFinder, the reference gene from the most to the least stability was ranked based on the stability value and the lowest stability value is the most stable.

### Validation of reference genes

*FaHY5* (ELONGATED HYPOCOTYL5), one of bZIP transcription factors was detected to validate the selected reference gene, which played an important role in plant development, signal transduction and stress response. The forward primer was 5ʹ-CAAGACCAAGCCACGAGC-3ʹ and the reverse primer was 5ʹ-TCCCTGCCTGAGACCGATG-3ʹ. Primer design and qRT-PCR reactions were followed as mentioned before. The most stable reference genes in different tissues and fruit developmental stages (HISTH4, DBP), light quality (GAPDH, DBP) and low temperature (ACTIN1, HISTH4) subgroups, and the least stable genes in different tissues and fruit developmental stages (ACTIN2, PIRUV), light quality (ACTIN2, PIRUV) and low temperature (ACTIN2, 18S) subgroups were used as the internal controls to calculate the expression level of *FaHY5*.

## Additional file

**Additional file 1: Figure S1.** PCR amplification of *FaACTIN1* on 2% agarose gel.
