# Supplementary figures and images for: Evaluation of suitable reference genes for qRT-PCR normalization in strawberry (Fragaria × ananassa) under different experimental conditions
Source: BMC Mol Biol. 2018 Jun 22;19:8. doi: 10.1186/s12867-018-0109-4 (PMC6013875; doi:10.1186/s12867-018-0109-4)

## Slide 1
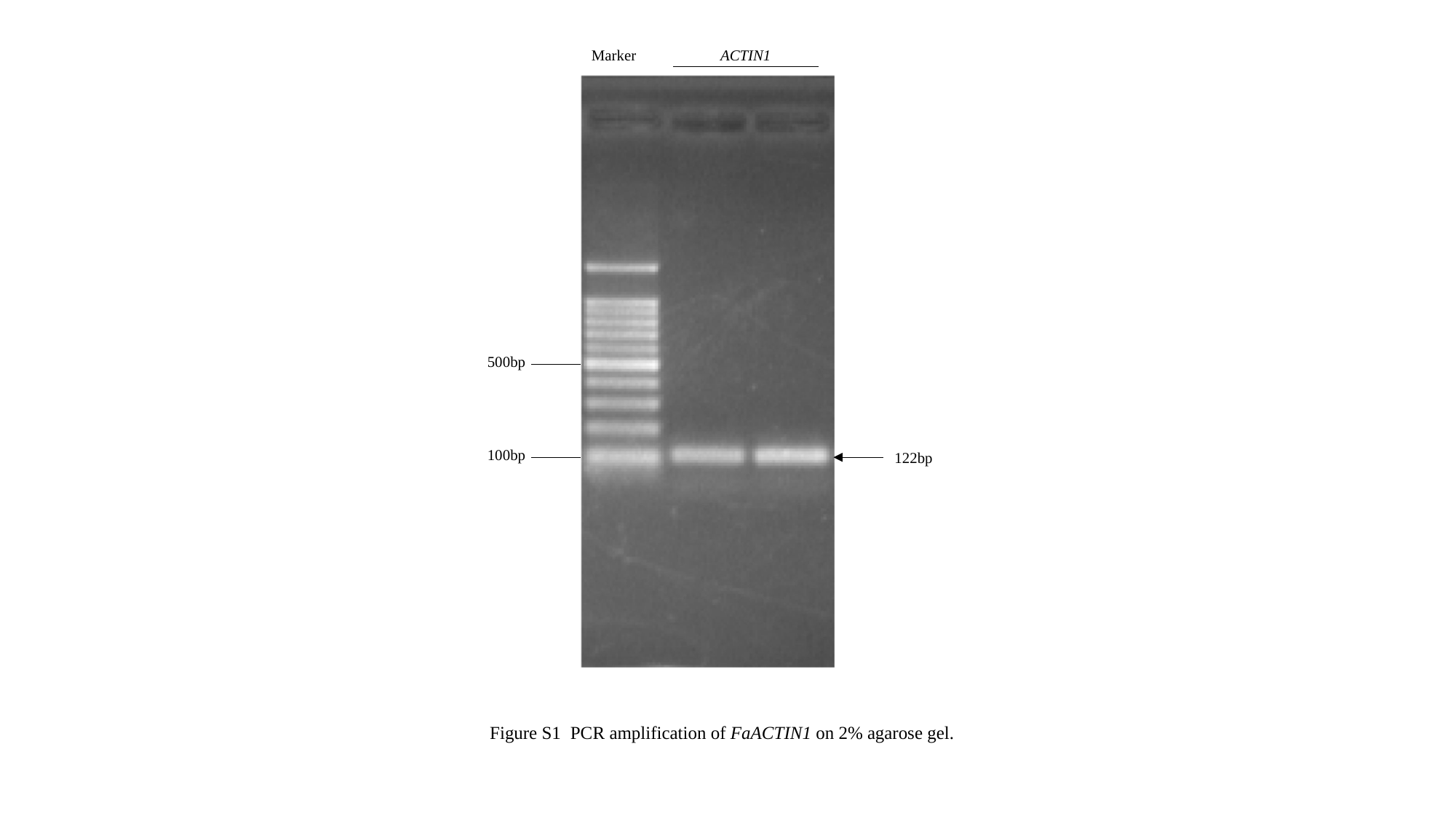

Marker
ACTIN1
500bp
100bp
122bp
Figure S1 PCR amplification of FaACTIN1 on 2% agarose gel.

Supplement: Supplementary file 1 — Additional file 1: Figure S1. PCR amplification of FaACTIN1 on 2% agarose gel. [file 12867_2018_109_MOESM1_ESM.pptx]
